# Attitudes of Patients With Chronic Heart Failure Toward Digital Device Data for Self-documentation and Research in Germany: Cross-sectional Survey Study

**DOI:** 10.2196/34959

**Published:** 2022-08-03

**Authors:** Lorina Buhr, Pauline Lucie Martiana Kaufmann, Katharina Jörß

**Affiliations:** 1 Department of Medical Ethics and History of Medicine University Medical Center Göttingen University of Göttingen Göttingen Germany; 2 Faculty of Economics, Law and Social Sciences University of Erfurt Erfurt Germany; 3 Department of Medical Informatics University Medical Center Göttingen University of Göttingen Göttingen Germany

**Keywords:** mobile health, mHealth, digital devices, wearables, heart failure, data sharing, consent, mobile phone

## Abstract

**Background:**

In recent years, the use of digital mobile measurement devices (DMMDs) for self-documentation in cardiovascular care in Western industrialized health care systems has increased. For patients with chronic heart failure (cHF), digital self-documentation plays an increasingly important role in self-management. Data from DMMDs can also be integrated into telemonitoring programs or data-intensive medical research to collect and evaluate patient-reported outcome measures through data sharing. However, the implementation of data-intensive devices and data sharing poses several challenges for doctors and patients as well as for the ethical governance of data-driven medical research.

**Objective:**

This study aims to explore the potential and challenges of digital device data in cardiology research from patients’ perspectives. Leading research questions of the study concerned the attitudes of patients with cHF toward health-related data collected in the use of digital devices for self-documentation as well as sharing these data and consenting to data sharing for research purposes.

**Methods:**

A cross-sectional survey of patients of a research in cardiology was conducted at a German university medical center (N=159) in 2020 (March to July). Eligible participants were German-speaking adult patients with cHF at that center. A pen-and-pencil questionnaire was sent by mail.

**Results:**

Most participants (77/105, 73.3%) approved digital documentation, as they expected the device data to help them observe their body and its functions more objectively. Digital device data were believed to provide *cognitive support*, both for patients’ self-assessment and doctors’ evaluation of their patients’ current health condition. Interestingly, positive attitudes toward DMMD data providing cognitive support were, in particular, voiced by older patients aged >65 years. However, approximately half of the participants (56/105, 53.3%) also reported difficulty in dealing with self-documented data that lay outside the optimal medical target range. Furthermore, our findings revealed preferences for the self-management of DMMD data disclosed for data-intensive medical research among German patients with cHF, which are best implemented with a dynamic consent model.

**Conclusions:**

Our findings provide potentially valuable insights for introducing DMMD in cardiovascular research in the German context. They have several practical implications, such as a high divergence in attitudes among patients with cHF toward different data-receiving organizations as well as a large variance in preferences for the modes of receiving information included in the consenting procedure for data sharing for research. We suggest addressing patients’ multiple views on consenting and data sharing in institutional normative governance frameworks for data-intensive medical research.

## Introduction

### Background

This study focused on views and attitudes toward the use of digital device data among patients with chronic heart failure (cHF) in Germany. cHF is one of the most prevalent health conditions in Western industrial societies, where morbidity and mortality rates are high [1]. Patients with cHF are among the patient groups with cardiovascular findings for whom digital self-documentation plays an increasingly important role in self-management. In terms of chronic cardiovascular diseases, German society may be considered a typical Western industrialized country. A key area of research currently focuses on reducing rehospitalizations, which are often associated with worsening syndrome progression [2,3]. In addition to pharmacological interventions and lifestyle changes, patient self-care and self-management are key factors in the overall treatment of the noncurable cHF. As part of cHF self-care, patient self-documentation or self-monitoring plays an important role because it allows the close and continuous monitoring of changes in different vital parameters to prevent possible readmission and allow timely countermeasures [4-11]. Self-documentation consists not only of regular self-monitoring of vital parameters, such as heart rate, blood pressure, and temperature, but also of recording physical activity or body weight [8,11]. In recent years, the use of digital mobile measurement devices (DMMDs) for self-documentation in cardiovascular care and research has increased in Western industrialized health care systems. This includes a range of devices for self-documentation, such as body scales or blood pressure monitors, mobile electrocardiograms, sensor devices, commercially available or medical-grade wearable technologies, and smartphone or tablet apps [12-14]. Throughout the text, we refer to the deployment of DMMDs for self-documentation as *digital self-documentation*, which we use synonymously with *digital self-monitoring*.

The digital self-documentation data of patients with cHF can be shared within telemonitoring programs and in data-intensive studies that collect and evaluate patient-reported outcome measures [15]. To do this, various vital signs are collected and transmitted for data analyses to remote health services, doctors, cardiology clinics, and research institutes. Preliminary evidence suggests that certain telemonitoring approaches have the potential to reduce hospitalization rates and improve the overall quality of life [3,14,16-20]. However, the implementation of data-intensive devices and data sharing pose several challenges for doctors and patients as well as for the ethical governance of data-driven medical research.

The main ethical challenges determined with the use of digital device data are data literacy and consent to the sharing of data gathered from DMMDs for health care and medical research. As per Koltay [21] and Johnson [22], data literacy may be defined as the “ability to process, sort and filter vast quantities of information, which requires knowing how to search, how to filter and process, to produce and synthesize it.” Concerning digital device data, the question that arises is to what extent patients have those abilities and how well they are able to analyze and handle their own digital health data. Regarding models and ways of consenting to participation in medical research, in recent years, a politically supported shift has emerged in Germany and other European countries contesting the standard model of *informed consent* [23-26] and propagating *broad consent* and *data donation* solutions [27-29]. Although *informed consent* aims to ensure that participants are enabled to make informed choices by disclosing all information about a study, that is, its specific purpose, research question, rationale, and risks, the *broad consent* model grounds on the reuse of patients’ data or biospecimens for various and rather unspecific research questions, aims, researchers, or studies [30-32]. We argue that the aforementioned challenges surrounding DMMD data require further ethical reflection on data-intensive medical research and cardiac care; for this in turn, a more patient-centered perspective is required [33-35].

### Previous Work

#### Attitudes Toward Sharing Digital Health Data for Research

In this paper, we present some work that has been carried out in Western industrialized countries, which also form—from a global perspective—the sociopolitical context for evaluating the German health care system and medical research. In the past decade, there has been an increasing number of qualitative, quantitative, and mixed methods studies in Western industrialized contexts that explored patients’ and users’ behaviors, attitudes, and perceptions regarding mobile phone–based health apps. These studies focused on wearable devices [36-39], health apps in general [40-42], and health apps for certain diseases, for example, mental health or chronic diseases [43,44]. Most of these studies aimed to identify facilitators and barriers to the uptake of wearables and apps, such as concerns regarding data security, privacy policies, and individual control over data [45-48]. There is, however, only limited literature concerning public and patients’ views on data practices and procedures within the scope of digital health self-documentation and data sharing for research purposes. The first systematic review of qualitative studies on these topics by Aitken et al [49] reported a general and widespread support for data sharing for research purposes among the public [50]. This depends, however, on the condition that respondents have trust in the individuals and research organizations that receive and analyze their data. These findings were strengthened by a systematic review study on the use of patient data for research in the United Kingdom and the Republic of Ireland carried out by Stockdale et al [51]. They found that the public “evaluates trustworthiness of research organizations by assessing their competence in data-handling and motivation for accessing the data.” A recent focus group study among patients with cardiac diseases in the Netherlands conducted by Wetzels et al [52] revealed that patients were not sufficiently informed about the aspects of data storage, data use, and access issues; furthermore, they “would prefer to have control over health data and to decide who be granted access and when.” Beierle et al [53] in their observation study also presented a rather complex picture of German smartphone users’ willingness to share their data; in addition to privacy concerns, personality traits, sex, and age were also found to be significant factors for refusing data sharing (N=461). In addition, according to a web-based survey of German students (N=682) and an analysis of data from the US Health Information National Trend Survey (N=2972-3155) by Kriwy and Glöckner [54], factors of self-declared poor health condition and a high level of education increased the willingness of patients to disclose device data on the web to their physicians or medical staff.

#### Consent Models for Data Sharing for Medical Research in Germany

Richter et al [55] conducted 4 seminal survey studies regarding consent models for sharing digital health data for research, in which they investigated attitudes toward broad consent and *no consent* policies in Germany (3 studies) and the Netherlands (1 study). The results of these studies are presented in 3 papers [29,55,56]. The first study was a delivery-and-collection questionnaire survey conducted between 2015 and 2016 in which 760 adult patients at an outpatient clinic for inflammatory conditions at the University Hospital Schleswig-Holstein, Campus Kiel were invited to participate. It focused on the comprehensibility of the provided broad consent form and informational brochure as well as motivations to agree to broad consent for health care–embedded biobanking [56]. This study design was repeated in 2018, inquiring into attitudes toward routine clinical care data for secondary use for scientific research without consent in line with the General Data Protection Regulation by the European Union (Regulation 2016/678G EU, EU-GDPR, §27; the final data set consisted of 503 patients) [29]. Both studies reported high willingness to provide a broad consent for hospital-based biobanking (661/760, 86.9%, and 468/503, 93%). In addition, the second study reported that three-fourths of the patients (381/503, 75.7%) supported a *no consent* regulation—sometimes called *data donation*—for medical data processing. This regulation is in accordance with the current German law under certain conditions [29]. Finally, a telephone-based population survey (N=1006) carried out by the Technology, Methods, and Infrastructure for Networked Medical Research and the German Forsa Institute in August 2019 in Germany largely confirmed these findings [55].

### Objectives

This study explored the potential and challenges of digital device data for cardiology research. Key questions concerned patients’ attitudes toward health-related data collected using DMMDs for self-documentation, sharing health data and consenting to data sharing. To address these questions, this study was conducted. The results can provide empirically based ethical recommendations for the future development and implementation of DMMD and consent solutions for data-intensive cardiology research. To our knowledge, no previous study has focused on the attitudes of patients with cHF toward sharing DMMD data for research.

## Methods

### Study Design

A cross-sectional survey of patients with cHF was conducted from March to July 2020 at the University Medical Center Göttingen (UMG). The survey was embedded in a wider comparative study that aimed to cover cardiovascular patients’ views and attitudes on DMMD data use. Considering the ongoing development of digital devices and mobile health apps in the domain of cardiovascular diseases, the questionnaire was neither device-specific nor app-specific and included diverse DMMDs in cardiovascular care and research. The survey study forms a substudy of the HiGHmed Use Case Cardiology (HiGHmed-UCC) project, an ongoing noninterventional, nonrandomized, multicenter registry study covering patients with cHF [57,58]. For HiGHmed-UCC, patients with cHF were recruited at the UMG. Patients were recruited either during routine visits to the heart failure outpatient department or during their hospitalization in the cardiology ward at the UMG. They provided informed consent to allow recall for further studies. This, in turn, was a condition for participating in the survey. The inclusion criteria for patients participating in our survey were those used for HiGHmed-UCC, that is, adults aged ≥18 years, German-speaking, diagnosed with cHF, capable of providing consent and expected to survive for >6 months, and consented to inclusion in HiGHmed-UCC.

### Ethics Approval

The HiGHmed-UCC and survey study were approved by the local Human Research Review Committee at the UMG (reference 21/9/18 and 28/7/18). For the survey study, no ethical and legal concerns were identified.

### Questionnaire and Survey Items

The survey questionnaire consisted of 66 questions or items. As a literature search for suitable questionnaires proved fruitless, we decided to construct a largely new questionnaire for our research purposes. The lack of suitable items, especially regarding attitudes toward self-documentation, digital devices, and digital device data, required de novo construction of 53 of 66 items specifically for this survey. The remaining 13 items were drawn from preexisting questionnaires or publications and modified for our purposes. Multimedia Appendix 1 [59,60] lists the items presented in this paper and the original versions of the modified items. Owing to the preponderance of nonvalidated, newly constructed items, we took the following measures to ensure the integrity of our questionnaire: during the questionnaire development process, survey items were repeatedly discussed within the HiGHmed ethics team in Göttingen and reviewed by Bioethics colleagues for comprehensibility and consistency. In addition, we conducted a pretest to improve the applicability of our questionnaire.

In the questionnaire, questions with one or multiple-choice options were included, and 6-point Likert scales for questions regarding patient attitudes were also included. This paper presents the results of items addressing the following topics: attitudes toward self-documentation and digital devices as well as self-documentation behavior and use of digital devices in daily life, attitudes toward digital device data and data sharing for research purposes along with data sharing conditions (modes of consent), attitudes toward medical research in general, and sociodemographic characteristics (age, gender, education, occupation, number of chronic diseases, and impairment due to diseases; Multimedia Appendix 1).

### Pretest

We conducted a pretest (N=11) with laypersons to check the general comprehensibility and feasibility of our questionnaire and detect potential problems with the items or questions included [61]. The age of the pretest participants ranged from 28 to 75 years (mean 60, SD 13). We included older adults to mirror the reality of most patients with cHF and cardiovascular diseases in Germany. On average, it took the pretest participants 32 minutes to complete the questionnaire. Suggestions for improvement and participants’ impressions regarding the comprehensibility and order of the items from the pretest were considered in the revision of the questionnaire.

### Recruitment and Sample

In the view of the ongoing global COVID-19 pandemic, eligible HiGHmed-UCC patients were contacted remotely by phone and informed regarding the survey and its purpose. We sent an information flyer and a questionnaire by mail to those who voiced their interest in participating. We tried to contact 190 patients, of whom 179 (94.2%) were finally approached. Of these 179 patients, 159 (88.8%) showed interest in our study and were sent the survey documents. Participants filled out the questionnaire at home. Overall, we received 108 completed questionnaires. Thus, a high level of participation was achieved (response rate: 67.9%). To participate in our survey, all participants had to provide a signed informed consent form containing a data protection declaration. Multimedia Appendix 2 provides an overview of recruitment and inclusion procedures.

### Statistical Analysis

Before conducting the statistical analysis, the 108 questionnaires were examined for completeness, and questionnaires with >30% missing data were excluded, which is an accepted cut-off mark in the literature [62]. After completing this examination, 105 questionnaires were included in the statistical analyses. We conducted descriptive statistics for all the items. Furthermore, we tested for differences in the attitudes toward self-documentation between sociodemographic groups. For the statistical analysis, age and subjective state of illness were grouped into binary categories. The age range was grouped into <65 and >65 years, drawing on the definition of a recent United Nations definition of *older persons* [63] and age for retirement in Germany. Subjective state of illness was grouped into mild (1-5 on a 10-point scale) and severe (6-10 on a 10-point scale). We carried out 2-tailed *t* tests to detect significant differences between the 2 groups. To detect inhomogeneity of variance, we conducted a Welch test. In cases lacking a normal distribution or in those where it could not be assumed owing to the size of the groups, we applied the nonparametric Mann-Whitney *U* test. To test for significant differences among >2 groups, we used the nonparametric Kruskal-Wallis test because the requirements for one-way ANOVA were not met. In this case, the Monte Carlo significance was reported. Post hoc testing was performed using the Dunn-Bonferroni test. Statistically significant differences between groups that showed no statistical significance after post hoc testing are not reported in this paper. Statistical significance was set at *P*<.05. All statistical analyses were performed using the SPSS software for Windows (version 26; IBM). Within the scope of this paper, we focused on descriptive analyses of the selected items dealing with the topics of DMMD data for self-documentation, research, and consent preferences.

## Results

### Sample Characteristics

The mean age of the participants was 65.12 (SD 10.952; range 35-85) years. Of the 104 patients, 76 (73.1%) were men and 28 (26.9%) were women. The largest number of respondents declared to have completed lower secondary school (41/105, 40%), followed by secondary school (24/105, 22.9%), higher secondary school examination (*Abitur*; 22/105, 20.9%), and advanced technical college entrance qualification (15/105, 14.3%). A small number (2/105, 1.9%) of participants dropped out of school. A total of 66.7% (70/105) of the participants had retired at the time of the study, 22.9% (24/105) were working, 6.7% (7/105) were homemakers, and 3.8% (4/105) declared an alternative occupation status. Regarding the number of chronic diseases, 38.5% (40/104) of the participants claimed to have 1 to 2 chronic diseases, 37.5% (39/104) reported 3 to 4, and 24% (24/104) reported ≥5. Almost half of the sample (45/105, 42.9%) disclosed mild disability owing to their disease, whereas the other half (60/105, 57.1%) experienced severe impairment in daily life. Table 1 provides an overview of the sociodemographic and health characteristics of the sample.

**Table 1:** Sociodemographic and health characteristics of the sample (n=104-105).^a^

Characteristic			Value, n (%)
**Gender (n=104)**			
	Female	28 (26.9)	
	Male	76 (73.1)	
**Age (years; n=105)**			
	<65	51 (48.6)	
	>65	54 (51.4)	
**Education (n=105)**			
	No education or dropout	2 (1.9)	
	Lower secondary school examination (Hauptschulabschluss)	41 (40)	
	Secondary school examination (Realschulabschluss)	24 (22.9)	
	Advanced technical college entrance qualification (Fachhochschulreife)	15 (14.3)	
	Final secondary school examination (Abitur or Hochschulreife)	22 (20.9)	
**Occupation (n=105)**			
	Working	24 (22.9)	
	Retired	70 (66.7)	
	Homemaker	7 (6.7)	
	Other	4 (3.8)	
**Chronic diseases (n=104)**			
	1 to 2	40 (38.5)	
	3 to 4	39 (37.5)	
	>5	24 (24)	
**Impairment from diseases (n=105)**			
	Mild	45 (42.9)	
	Severe	60 (57.1)	

^a^Variance in the sample set was due to incomplete person-related data.

### Attitudes Toward Self-documentation and Device Data

Half of the participants reported performing self-documentation (53/105, 50.5%), and 55.2% (58/105) of the participants were using a digital device at the time of the survey. One-third (16/46, 35%) of the patients who did not use a digital device at the time of the survey had previously tried using a device.

**Figure 1:**
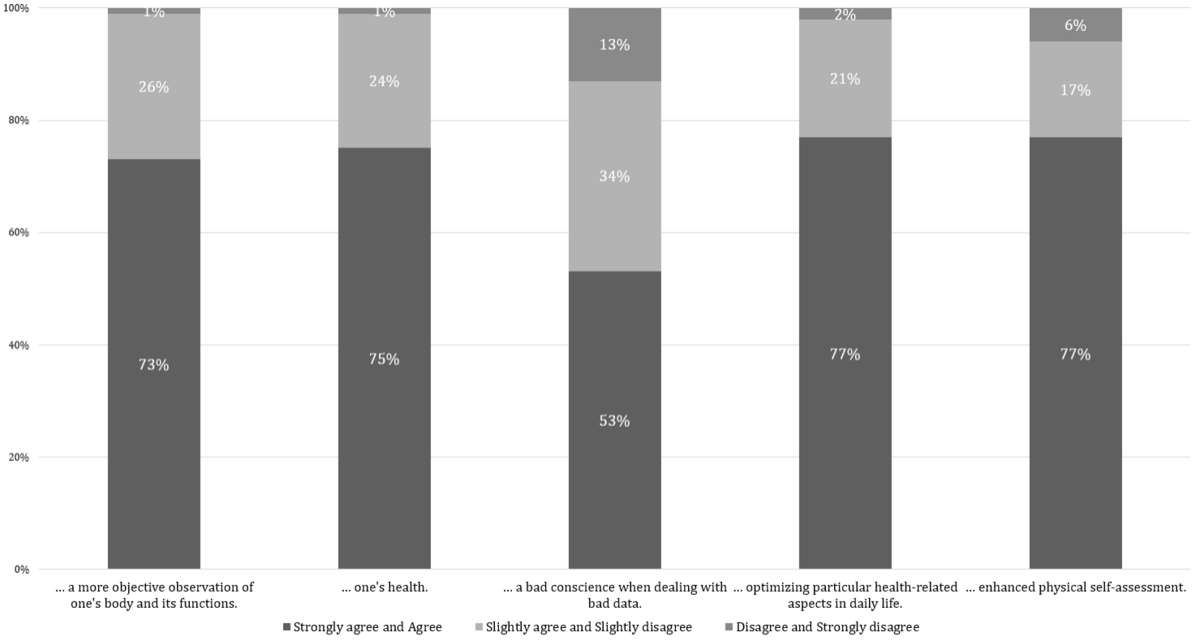
Attitudes toward self-documentation (n=105).

In terms of general attitudes toward self-documentation, 73.3% (77/105) of the participants stated that self-documentation helps in observing the body and its functions more objectively. Moreover, 77.1% (81/105) of the participants felt that self-documentation enhanced their overall physical self-assessment. The vast majority (79/105, 75.2%) of the participants found self-documented data to be health promoting, and 77.1% (81/105) of the participants stated that it helped to optimize health-related aspects of daily life. Approximately half of the participants (56/105, 53.3%) reported discomfort when confronted with self-reported data that lay outside the optimal medical target ranges. Figure 1 provides an overview of the results. The main reasons for digital self-reporting by survey participants were as follows: 54% (31/58) wanted to improve their health, 45% (26/58) wished to provide health-related data for their doctors, 41% (24/58) required health-related data for themselves, and 40% (23/58) sought a better understanding of their body and its functions. In general, most participants (80/105, 76.2%) assumed that DMMD data would help doctors better understand their patients.

### Factors Influencing Attitudes Toward Self-documentation

Statistical analysis showed significant differences between younger and older participants regarding 4 of the 5 items that addressed general attitudes toward self-documentation. Older participants (aged >65 years) considered that self-documentation aided observing the body and its functions (*P*=.006), enhancing overall physical self-assessment *(P*=.001), promoting health (*P*=.008), and optimizing certain health-related aspects in daily life (*P*=.03; Multimedia Appendix 3). No statistical significance was found between younger and older participants regarding negative emotions when dealing with self-documented data that lay outside the optimal medical target ranges (*P*=.41). Further statistical analysis revealed no statistically significant differences in terms of sociodemographic groups of gender, chronic diseases, and impairment.

### Attitudes Toward Sharing Device Data for Research

#### Characteristics of Data Sharing and Consenting

First, the overwhelming majority of participants (99/105, 94.3%) expressed a positive attitude toward medical research. When asked about concerns regarding personal DMMD data use in medical research, one-third of the participants feared data leakage (33/103, 32%) or its abuse (39/104, 37.5%). Most participants (65/104, 62.5%) believed that data protection regulations provided by the current German law were adequate. Nonetheless, the anonymization of personal digital device data was deemed important by the vast majority (87/104, 83.7%) of participants. In terms of consent, 83.7% (87/104) of the participants considered one-time information or education about sharing DMMD with medical research sufficient. By contrast, only 54.8% (57/104) of the participants considered receiving general information about the respective aims of medical research without detailed information about individual research projects sufficient. Most participants wanted to access shared digital device data (79/105, 75.2%) as well as have the option to delete some or all of the shared data (71/104, 68.3%). More than half of the participants (62/104, 59.6%) could envisage nonprofit organizations assuming the management of their shared digital device data. Few participants (16/104, 15.4%) feared discrimination due to research findings to which they had contributed.

#### Strong Difference Between State-Funded and Private Organizations

Participants were asked whether they would agree to share their data with various organizations and actors. Almost all participants approved sharing data with their family doctors (99/105, 94.3%) and state-run research institutions (97/105, 92.4%), whereas only 33.3% (34/102) of the participants agreed to share data with private research institutions, and 33% (34/103) of the participants agreed to share data with collaborative projects involving private corporations and state-run research institutions. Only few participants (17/101, 16.8%) would share DMMD data with public authorities. Just over a third (36/102, 35.3%) of the participants would share their DMMD data with public health insurance companies, whereas only 23.1% (24/104) of the participants would share the same data with private health insurance companies. Remarkably, few participants (13/102, 12.8%) agreed to share their digital device data with smaller companies, and even fewer participants (9/103, 6.7%) agreed to share their digital device data with large international companies. Multimedia Appendix 4 presents the summary statistic on attitudes toward data-receiving organizations and actors.

## Discussion

### Principal Findings and Comparison With Previous Work

This study examined the attitudes of a sample of patients with cHF in Göttingen, Germany, toward digital self-documentation and sharing of DMMD data with research institutes. Here, we focus on 3 key study findings. First, the results showed positive attitudes overall toward self-documentation among patients with cHF. Second, there were high expectations of DMMD data provision, which we propose to call *cognitive support* for the patients and for doctors to improve understanding of their patients’ health conditions. Third, the findings indicated a range of preferences and needs in terms of features and requirements for consent in the context of sharing DMMD data for research.

### Affirmative Attitudes but Also Emotional Stress Toward Self-documentation in Case of Irregular Data

Notably, most participants (74/105, 70.5%) had experience of digital self-documentation, either formerly (16/105, 15.2%) or at the time of the survey (58/105, 55.2%). This indicates a widespread openness toward conducting digital self-documentation among the patients with cHF surveyed. In support of this finding, our study showed an overall positive attitude toward self-documentation, with three-fourths (79/105, 75.2%) of the participants stating that self-documentation would promote one’s health and help to optimize health-related aspects of daily life (Figure 1).

As one-third of the patients (16/46, 35%) not performing digital self-documentation at the time of the survey had given up previous DMMD use, the potential of digital self-documentation turned out to be limited accordingly. Interestingly, for just over half of the participants yielding health data outside the normal range, this was accompanied by worries leading to mental and emotional stress. Statistical analysis showed no significant difference between younger and older participants in this respect. This finding suggests that negative feelings due to irregular data potentially affects all patients. Our result is consistent with those of other studies reporting that the negative mental impact of *abnormal data* can accompany device use [64,65]. Thus, digital self-documentation can potentially pose a significant burden for self-care [66]. Sjöklint et al [67] found that emotional tensions occurring due to reflecting on personal device data may promote neglect of device use and even induce its complete rejection. As approximately half of the patients with cHF experienced emotional stress, this poses a considerable challenge for DMMD use.

### Digital Self-documentation Data as Cognitive Support for Patients and Doctors

Our results reveal further interesting aspects. Many of our participants not only had high expectations of health promotion but also believed that self-documentation could enhance their knowledge base for understanding (77/105, 73.3%) and assessment of their own bodies and health conditions (81/105, 77.1%). Thus, data-intensive self-documentation was ascribed as *cognitive support*. As we had no items that asked for what we term *cognitive support*, it is a concept that we introduced when we interpreted the collected data from our survey. The effect of cognitive support, as we understand it, was considered to serve patients by increasing self-understanding and improving self-assessment and the doctor-patient relationship owing to an enlarged database. It is also striking that almost half of the patients conducting digital self-documentation stated that they did so to provide health-related data for their attending doctors. A possible explanation for this might be that these patients consider DMMD data to provide doctors with more precise information about their physical condition, thus improving their quality of care. These results are consistent with those of Tran et al [39], who also found that many patients believed that the use of biometric monitoring devices would improve caregivers’ work (21%) and communication (17%). Our statistical analysis showed that especially older and retired participants considered self-documentation and device data valuable for self-assessment and self-understanding and thus offered cognitive support. This is surprising because older people are often reported to need detailed training and intensified support when dealing with new digital technologies [68-70]. Against this backdrop, our findings indicate a gap between actual digital device use and public perceptions of device users. Thus, further research is needed to demonstrate how older people engage with and use personal DMMD data in their daily lives. Regarding cognitive support for patients’ self-understanding and self-assessment, this is a remarkable finding, as relying on device data for self-assessment requires the ability to interpret and handle these data. Self-assessment via DMMD data needs, in other words, data literacy and, in the case of digital self-documentation, the advanced skill of eHealth literacy. Future research should investigate whether patients’ eHealth literacy correlates with the expectation that self-documentation provides cognitive support. To measure eHealth literacy in the context of DMMD use for cHF treatment and prevention, the *eHealth Literacy Scale* developed by Norman and Skinner [71] seems to be a promising option (eg, the patient survey study by Knitza et al [72] in rheumatology by using the validated German version of eHealth Literacy Scale [73]).

### Heterogeneous Preferences for Data Sharing With Research

To identify attitudes toward sharing data from digital self-documentation for research, four aspects warrant consideration: (1) concerns about sharing data, (2) preferred modalities of data sharing and transmission, (3) informational conditions for consent, and (4) preferences for bodies receiving and mediating device data. The last 3 aspects present crucial dimensions for consenting to data sharing for research.

### Concerns About Data Sharing With Research

Our findings revealed a positive attitude toward medical research in general. However, there were some concerns about sharing data for research, as approximately one-third of the participants feared data leakage or abuse. Furthermore, some participants (16/104, 15.4%) feared discrimination when DMMD data are disclosed. By contrast, almost two-thirds of the participants (65/104, 62.5%) accepted the current legal data regulations as sufficient. Other studies showed that, generally, there seems to be widespread support for data sharing for research [55]. Trust in research organizations and data protection regulations as well as possible public benefits from research mostly outweigh concerns regarding data security and privacy [49]. Our study confirms these findings. Although there were data security concerns, trust in medical research and data protection regulations was high. Therefore, it is necessary for research organizations to consolidate public trust by adequately addressing concerns such as data abuse, leakage, and potential discrimination [74]. This should be considered when engaging with potential participants in a data sharing research project.

### Preferred Modalities of Data Sharing and Management

Regarding the preferred modalities of data sharing for research, attitudes were less heterogeneous, as for the vast majority of participants anonymization, having access to disclosed data, and the option to delete DMMD data were priorities. In addition, the majority of participants (62/104, 59.6%) approved management of DMMD data via a nonprofit organization. Thus, although only some participants (4/105, 3.8%) disagreed to share their DMMD data for research, most participants (62/104, 59.6%) approved such an *intermediate mode* of institutional data disclosure with research institutions. This would allow retaining the control and management of device data, either by patients themselves or by a nonprofit organization. On the basis of these findings, we can infer that patients with cHF favor a controlled mode of data sharing with options to manage disclosed data continuously and confidentially. This interpretation is also consistent with the results of the focus group study by Wetzels et al [52].

### Informational Requirements of Consenting

Turning now to preferred solutions for providing information on research that would receive and use disclosed data, we again obtained a heterogeneous picture. On the one hand, for the vast majority of participants (87/104, 83.7%), one-time provision of information about sharing device data for research was considered sufficient. On the other hand, only half of the participants (57/104, 54.8%) considered receiving general information about the respective purposes of medical research sufficient. The apparent inconsistency of these results can be resolved if we interpret this finding as a widespread preference for a one-time instruction about the actual data sharing procedures for research combined with mixed attitudes toward the provision of detailed information on specific research projects and their aims. As the broad consent model for data sharing in medical contexts rests on the principle of general, not detailed, information provision on research aims, it is striking to note that almost half of the patients with cHF in this study tended to disagree with the broad consent model. This outcome conflicts with the results of Richter et al [29] who reported a very high willingness (436/468, 93%) to give broad consent for health care–embedded biobanking among outpatients in an inflammatory disease clinic in Germany. A possible explanation for this might be that patients with cHF are more wary of the management of large-scale health data than those with diseases not subject to data-intensive monitoring.

### Preferences on Data-Receiving Organizations

The fourth aspect of data sharing relates to attitudes toward organizations that receive data. One important finding was the extent to which attitudes toward state-funded and private research organizations vary among participants in this study: private research institutions and collaborative research projects combining publicly funded and private organizations (*public-private partnerships*) were considerably less endorsed for the sharing of device data. Here, we interpret a preference for an organization as an expression of trust. We found that trust in state-funded research institutes as well as in physicians is very high (>90% participants). This is an encouraging message for state-funded research intuitions despite ongoing public debate on privacy and data security. However, the large gap between state-funded and private research institutes, collaborative research projects, and private companies poses a challenge for mobile device development, which is mainly performed in public-private partnership consortia. Our findings corroborate those of Aitken et al [49], Stockdale et al [51], and Richter et al [55]. For example, a study of the population survey by Richter et al [55] reported a striking difference in willingness to share health data anonymously and free of charge with university and public research institutions on the one hand (96.7%) and with privately funded research institutes and industry for research purposes (16.6%) on the other hand.

### Limitations

Several limitations of this study should be acknowledged, notably those affecting sampling. There is always a chance of latent bias from the underrepresentation of certain subgroups when opting for convenience sampling, as we did [75]. We observed a higher percentage of male participants (76/105, 73.1%) as opposed to the more even gender distribution of patients with cHF in Germany [76]. Two-thirds of the participants (70/105, 66.7%) already had some experience with DMMDs. Studies have reported technical affinity and male gender as facilitators for the use of self-documentation devices [59,70]. This could explain the high rate of DMMD experiences among male participants, as technologically savvy males might have been more likely to respond to our survey. In addition, although this is not statistically significant, their experiences might have positively colored their views on self-documentation. Furthermore, our participants formed part of a uniform group consisting of patients with cHF treated at the UMG, and all the participants were already part of the HiGHmed-UCC. Those interested in digital devices and data sharing may have participated more readily. In addition, the homogeneity and limited size of our sample make it difficult to perform inferential statistical analysis, given the possible departures from a normal distribution. It is noteworthy that attitudes reported in our study do not necessarily translate into future patient behavior when dealing with self-documentation, digital devices, and opportunities for sharing digital device data. Concepts of health conditions, types of data sharing, and research modalities are notoriously difficult to convey to a lay population, leaving room for potential misunderstandings when answering our survey questionnaire. Finally, our survey was limited to fluent German speakers, which might have further reduced the sample diversity. Despite its limitations, our study provides new insights into our understanding of attitudes of patients with cHF toward digital self-documentation and sharing device data for research as well as raises questions to be addressed in future studies in the German context. However, caution is required given the sample size limitations and any potential bias inherent in the study design; the findings might not be widely applicable to all patients with cHF or cardiovascular diseases.

### Conclusions

#### Overview

The rapidly expanding field of digital devices in cardiac health care and research needs to engage with the attitudes and perceptions of patients and probands [33-35]. Current device development is accompanied by governance policies and research on ethical, legal, and social issues (ELSI). These frameworks consider the privacy and data safety perceptions of the broad population as key issues. Our survey study focused on the potential of digital self-documentation and sharing device data for data-intensive research among patients with cHF at a German university medical center. The results showed that self-documentation and device data play a major role in supporting self-care in patients with cHF. The survey study was conducted with a rather limited sample size; 190 patients were originally approached and 105 questionnaires were included in the statistical analysis. Recruitment was considerably limited owing to the pandemic situation in Germany in 2020. As we achieved a very high rate of survey participation (67.9%), the results, however, have good significance for the sample of patients with cHF at the university medical center. However, owing to sample size limitations and potential bias inherent in the study design, limitations in the general applicability of these results must be considered. Nevertheless, our findings provide valuable insights for introducing DMMD into cardiovascular research in the German context. Furthermore, although our findings result from a restricted sample of patients with cHF at a clinic in Germany, they might also contribute to a large-scale cross-cultural and cross-national comparative study on views of patients with cardiovascular diseases on data-driven methods and technology deployment, which is still a considerable research goal. In general, more research is needed on the specificities of data-intensive research methods and technology across Western industrialized countries and countries of the global south. In any case, the results of our survey study among German patients with cHF have many practical implications for the German context, as detailed in the following sections.

#### Practical Implications for Doctors

First, doctors should become aware that many patients with cHF endorse sharing DMMD data with their family doctors. For these patients, it might be disappointing should their doctors refuse to engage with DMMD data for cognitive support. Second, for older patients with cHF, self-documentation data played a crucial role in self-assessment. Accordingly, they might be more open-minded toward digital self-documentation than is commonly supposed. Third, our findings indicate that the handling of problematic data warrants special consideration in the introduction and use of the devices in cardiovascular treatment.

#### Practical Implications for the Implementation of Data Sharing for Research

Our findings have significant implications for the implementation of technical solutions and governance models for data sharing and consent in cardiac research in Germany. First, our study documents at least two types of attitudes among patients with cHF regarding concerns raised by practices of data sharing in medical contexts: those who widely rely on current data protection regulations (this was the majority) and those who raise serious concerns about data security, misuse, and potential discriminatory effects when data are disclosed. From an ethical standpoint, these concerns should be addressed in communication and information procedures as well as in the technical and normative governance structures of data sharing in medical contexts. The same applies equally to, and this is the second implication, the preferred consent models in practice. The results of our study showed preferences for a dynamic rather than a broad consent approach among our survey participants with cHF. The dynamic consent model allows participants to handle permissions, education, and consent preferences in data-intensive medical research *dynamically* by selecting and modifying consent options temporally via digital consent tools [32,77-79]. Collectively, our findings provide key insights for the design of data sharing programs and data-intensive research projects in cardiovascular research and care at clinics and university medical centers in Germany.

## Appendix

Multimedia Appendix 1 Topics and items of the questionnaire reported in this paper.

Multimedia Appendix 2 Flowchart of sample recruitment.

Multimedia Appendix 3 Correlations between age of the participants and attitudes toward self-documentation.

Multimedia Appendix 4 Preferences on data-receiving organizations (n=101-105).

## Abbreviations

cHF: chronic heart failure
DMMD: digital mobile measurement device
ELSI: ethical, legal, and social issues
HiGHmed-UCC: HiGHmed Use Case Cardiology
UMG: University Medical Center Göttingen

## Data Availability

The data sets generated and analyzed during this study can be obtained from the corresponding author upon reasonable request.
