# Supplementary material for: Attitudes of Patients With Chronic Heart Failure Toward Digital Device Data for Self-documentation and Research in Germany: Cross-sectional Survey Study
Source: JMIR Cardio. 2022 Aug 3;6(2):e34959. doi: 10.2196/34959 (PMC9386578; doi:10.2196/34959)
Supplement: Multimedia Appendix 1 [file cardio_v6i2e34959_app1.pdf]

Multimedia Appendix 1

Topics and items of the questionnaire reported in this paper (original German titles)

Item no. in questionnaire	Item	Answer	Origin of the Item
	**(Digital) Self-Documentation**		
2.1	Betreiben Sie zurzeit irgendeine Form der Selbstdokumentation?	Yes/No.	own
2.2	Haben Sie in der Vergangenheit irgendeine Form der Selbstdokumentation betrieben?	Yes/No.	own
2.3	Selbstdokumentation hilft, den Körper und seine Funktionen sachlich betrachten zu können.	6-point-Likert-scale[a]	own
2.4	(Selbstdokumentation und die erhobenen Daten sind förderlich für die eigene Gesundheit.	6-point-Likert-scale[a]	own
2.5	Wenn sich bei der Selbstdokumentation Daten zeigen, die nicht im gesundheitlich optimalen Bereich liegen, führt das manchmal zu einem schlechten Gewissen	6-point-Likert-scale[a]	own
2.6	Selbstdokumentation hilft, bestimmte gesundheitsbezogene Aspekte im Leben zu verbessern.	6-point-Likert-scale[a]	own
2.7	Selbstdokumentation verbessert die körperliche Selbsteinschätzung.	6-point-Likert-scale[a]	own
	**Use of Digital Measurement Devices (DMMD)**		
2.8	Benutzen Sie zurzeit ein solches Gerät/mehrere solcher Geräte im Alltag?	Yes/No.	own
2.9	Haben Sie in der Vergangenheit ein solches Gerät/mehrere solcher Geräte benutzt?	Yes/No.	own
2.14	Ich nutze das digitale, mobile Messgerät/die Messgeräte, weil …	Multiple answers are possible:<br><br>ich mich dazu motivieren möchte, meinen Gesundheitsstatus zu verbessern oder zu halten.<br><br>ich ein besseres Verständnis für meinen Körper und seine Funktionen erreichen möchte.<br><br>ich meine gesundheitsbezogenen Daten für mich dokumentieren möchte.<br><br>es für mich ein Statussymbol darstellt.<br><br>ich meine gesundheits-bezogenen Daten für meine*n Ärzt*in dokumentieren möchte.<br><br>ich an einer Studie zu Messgeräten teilnehme.<br><br>Kein besonderer Grund.	own, as well as [59]

Multimedia Appendix 1

Topics and items of the questionnaire reported in this paper (original German titles)

		Sonstiges.	
2.18	Die Informationen über Körperwerte, die ein digitales, mobiles Messgerät sammelt, helfen Ärzt*innen, die Patient*innen besser zu verstehen.	6-point-Likert-scale[a]	own
	**Sharing Device Data (for Research)**		
3.2	Ich bin besorgt, dass die Daten im Rahmen eines sogenannten Datenlecks [=unerlaubter Zugriff auf vertrauliche Daten] in die Öffentlichkeit gelangen.	6-point-Likert-scale[a]	own
3.3	Ich mache mir Gedanken um einen möglichen Missbrauch der Daten durch Dritte.	6-point-Likert-scale[a]	own
3.4	Wenn ich neuartige Erkenntnisse zu meiner Krankheit erlangen will, bin ich darauf angewiesen, dass die Daten von Forschungseinrichtungen analysiert werden.	6-point-Likert-scale[a]	own
3.11	Ich kann mir gut vorstellen, einer gemeinnützigen Organisation die Verwaltung der Daten für die medizinische Forschung anzuvertrauen.	6-point-Likert-scale[a]	own
3.13	In welcher Form sollen die Daten vorliegen, die Sie der medizinischen Forschung zur Verfügung stellen würden?	Multiple answers are possible:<br><br>Identifizierende, nicht verschlüsselte Daten.<br><br>Nur anonymisierte Daten (Sie könnten nicht mehr identifiziert werden.)<br><br>(Pseudonymisierte Daten (Sie als betroffene Person könnten nur mithilfe eines Kodes identifiziert werden).<br><br>Es ist mir egal, in welcher Form die Daten vorliegen und an die Forschung weitergegeben werden würden.<br><br>Ich würde keine Daten zur Verfügung stellen.	own
3.14	Welche der mithilfe eines digitalen, mobilen Messgerätes erhobenen und pseudonymisierten Daten<br>würden Sie der medizinischen Forschung zur Verfügung stellen?	Yes/No/ I do not know.	own
3.15	Ich bin damit einverstanden, dass an mir mithilfe eines digitalen, mobilen Messgerätes erhobene und pseudonymisierte Daten weitergegeben werden an Hausärzt*in.	Yes/No/ I do not know.	own
3.16	Ich bin damit einverstanden, dass an mir mithilfe eines digitalen, mobilen Messgerätes erhobene und pseudonymisierte Daten weitergegeben werden an Staatliche Forschungseinrichtungen [z.B. Universitäten].	Yes/No/ I do not know.	own
3.17	Ich bin damit einverstanden, dass an mir mithilfe eines digitalen, mobilen Messgerätes erhobene und	Yes/No/ I do not know.	own

Multimedia Appendix 1

Topics and items of the questionnaire reported in this paper (original German titles)

	pseudonymisierte Daten weitergegeben werden an Private Forschungseinrichtungen.		
3.18	Ich bin damit einverstanden, dass an mir mithilfe eines digitalen, mobilen Messgerätes erhobene und pseudonymisierte Daten weitergegeben werden an Ämter und Behörden.	Yes/No/ I do not know.	own
3.19	Ich bin damit einverstanden, dass an mir mithilfe eines digitalen, mobilen Messgerätes erhobene und pseudonymisierte Daten weitergegeben werden an Öffentliche Krankenkassen.	Yes/No/ I do not know.	own
3.20	Ich bin damit einverstanden, dass an mir mithilfe eines digitalen, mobilen Messgerätes erhobene und pseudonymisierte Daten weitergegeben werden an Private Krankenkassen.	Yes/No/ I do not know.	own
3.21	Ich bin damit einverstanden, dass an mir mithilfe eines digitalen, mobilen Messgerätes erhobene und pseudonymisierte Daten weitergegeben werden an Kleine bis mittelständige Unternehmen mit Sitz in Deutschland.	Yes/No/ I do not know.	own
3.22	Ich bin damit einverstanden, dass an mir mithilfe eines digitalen, mobilen Messgerätes erhobene und pseudonymisierte Daten weitergegeben werden an Internationale Großunternehmen.	Yes/No/ I do not know.	own
3.23	Ich bin damit einverstanden, dass an mir mithilfe eines digitalen, mobilen Messgerätes erhobene und pseudonymisierte Daten weitergegeben werden an Forschungsprojekte, die staatliche Einrichtungen in Zusammenarbeit mit Wirtschaftsunternehmen durchführen.	Yes/No/ I do not know.	own
	**Consent for Data Sharing**		
3.5	Ich bin davon überzeugt, dass die Daten durch gesetzlich vorgeschriebene Maßnahmen ausreichend geschützt sind.	6-point-Likert-scale[a]	own
3.6	Mir ist die Anonymisierung der Daten für den Gebrauch in der Forschung wichtig. Hinweis: Anonymisierung ist eine Datenschutzmaßnahme, die dafür sorgt, dass Ihre Daten nicht mehr Ihrer Person zugeordnet werden können.	6-point-Likert-scale[a]	own
3.7	Ich finde es ausreichend, einmalig über die Weitergabe der Daten an die medizinische Forschung aufgeklärt zu werden.	6-point-Likert-scale[a]	own
3.8	Ich finde es ausreichend, über die Weitergabe der Daten an die medizinische Forschung ohne Benennung bestimmter Forschungsprojekte aufgeklärt zu werden.	6-point-Likert-scale[a]	own
3.9	I would like to reserve the right to access the data at any time.<br><br>(Ich möchte mir vorbehalten, jederzeit auf die Daten zugreifen zu können.)	6-point-Likert-scale[a]	own
3.10	Ich möchte mir vorbehalten, jederzeit alle oder einzelne Daten löschen zu können.	6-point-Likert-scale[a]	own

Multimedia Appendix 1

Topics and items of the questionnaire reported in this paper (original German titles)

	**Socio-demographic and personal characteristics and attitudes**		
4.1	Ich habe eine positive Grundeinstellung gegenüber medizinischer Forschung.	6-point-Likert-scale[a]	Modified after [60]
5.1	Welches Geschlecht haben Sie?	Frau/Mann/Divers	own
5.2	Wie alt sind Sie?	open	own
5.3	Welche Schulbildung haben Sie? Bitte den höchsten Abschluss angeben	Keinen Abschluss<br>Hauptschule/Volksschule<br>Realschule/Mittlere Reife<br>Fachhochschulreife<br>Abitur/allgemeine Hochschulreife	own
5.4	Zurzeit sind Sie …	Single choice:<br>Berufstätig<br>Hausmann/*frau<br>Berentet<br>Schüler*in, Student*in, Auszubildende*r<br>Arbeitslos/erwerbslos<br>Sonstiges	own
5.5	Wie viele dauerhafte [chronische] Krankheiten haben Sie?	1-2<br>3-4<br>>5	own
5.6	Wie beeinträchtigt fühlen Sie sich in Ihrem Alltag durch Ihre Krankheit/en?	10-point-Likert-scale[b]	own

[a] Likert-scale: 1: I completely agree, 6: I entirely disagree

[b] Likert-scale: 1: not impaired at all, 10: very strongly impaired
