# Supplementary material for: Attitudes of Patients With Chronic Heart Failure Toward Digital Device Data for Self-documentation and Research in Germany: Cross-sectional Survey Study
Source: JMIR Cardio. 2022 Aug 3;6(2):e34959. doi: 10.2196/34959 (PMC9386578; doi:10.2196/34959)

Multimedia Appendix 2

Flow chart: recruitment and sample

Aim: Cross-sectional survey based on a sample of 100 participants.

Inclusion criteria/ population sample: (1) full age (≥18 years), (2) German speaking, (3) diagnosed with chronic heart failure, (4) capable of giving consent and being expected to survive more than 6 months, (5) having consented to inclusion in HiGHmed-UCC

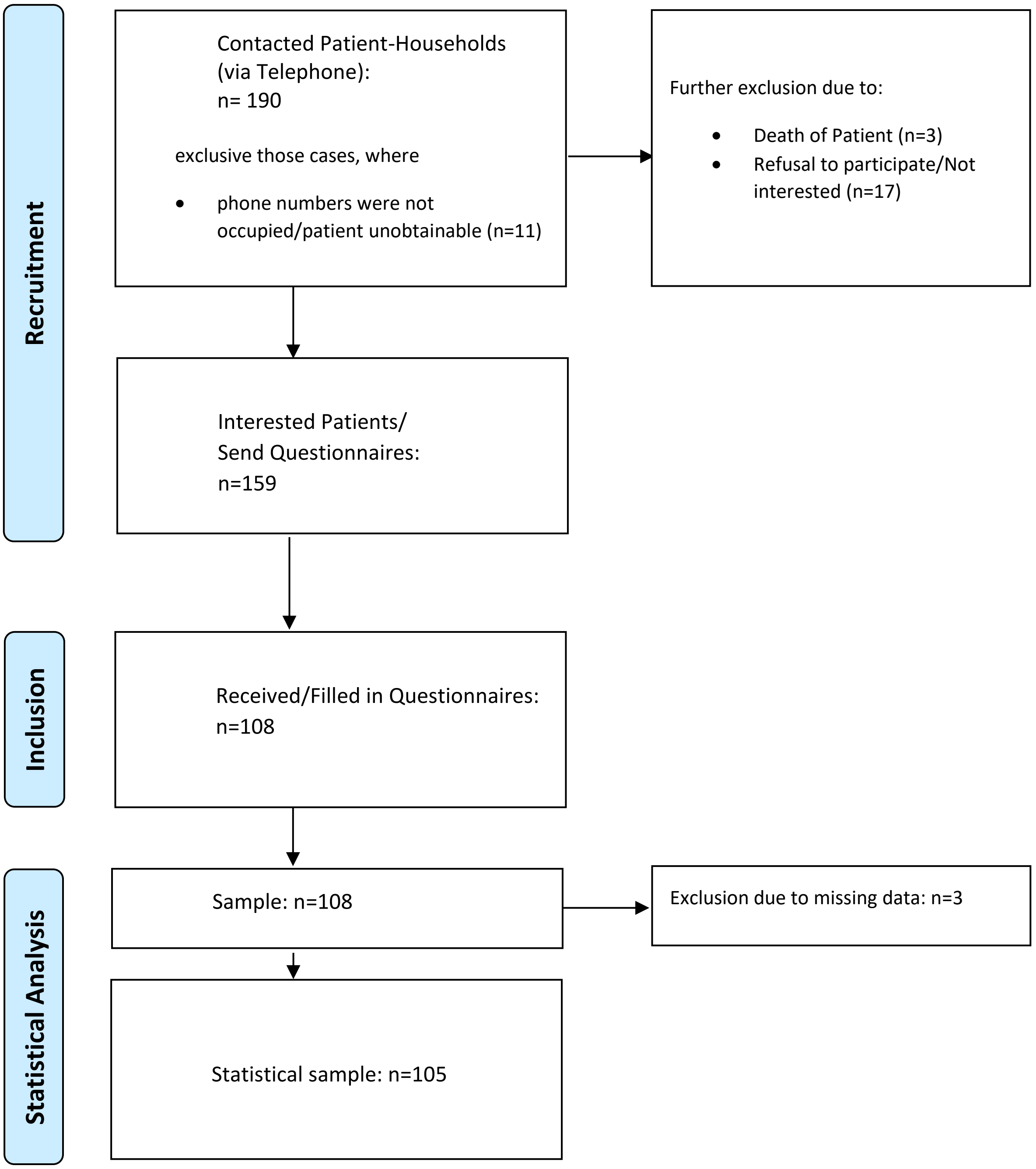

Supplement: Multimedia Appendix 2 [file cardio_v6i2e34959_app2.pdf]
