# Supplementary material for: Attitudes of Patients With Chronic Heart Failure Toward Digital Device Data for Self-documentation and Research in Germany: Cross-sectional Survey Study
Source: JMIR Cardio. 2022 Aug 3;6(2):e34959. doi: 10.2196/34959 (PMC9386578; doi:10.2196/34959)
Supplement: Multimedia Appendix 3 [file cardio_v6i2e34959_app3.pdf]

Supplementary Material 3

Correlations between age of participants and attitudes towards self-documentation (6-point-Likert scale)

Item no. in questionnaire	Item	Test	Age <65 (n=51) [mean (SD)]	Age ≥ 65 (n=54) [mean (SD)]	*P* value
	**(Digital) Self-Documentation**				
**2.3**	Selbstdokumentation hilft, den Körper und seine Funktionen sachlich betrachten zu können.	independent t-test	2.24 (1.142)	1.69 (0.843)	.006
**2.4**	Selbstdokumentation und die erhobenen Daten sind förderlich für die eigene Gesundheit.	independent t-test	2.16 (1.007)	1.65 (0.935)	.008
**2.5**	Wenn sich bei der Selbstdokumentation Daten zeigen, die nicht im gesundheitlich optimalen Bereich liegen, führt das manchmal zu einem schlechten Gewissen	independent t-test	2.56 (1.458)	2.81 (1.333)	.408
**2.6**	Selbstdokumentation hilft, bestimmte gesundheitsbezogene Aspekte im Leben zu verbessern.	independent t-test	2.12 (1.125)	1.7 (0.792)	.031
**2.7**	Selbstdokumentation verbessert die körperliche Selbsteinschätzung.	Welch’s t-test	2.37 (1.341)	1.63 (0.73)	.001
