# Supplementary figures and images for: Attitudes of Patients With Chronic Heart Failure Toward Digital Device Data for Self-documentation and Research in Germany: Cross-sectional Survey Study
Source: JMIR Cardio. 2022 Aug 3;6(2):e34959. doi: 10.2196/34959 (PMC9386578; doi:10.2196/34959)

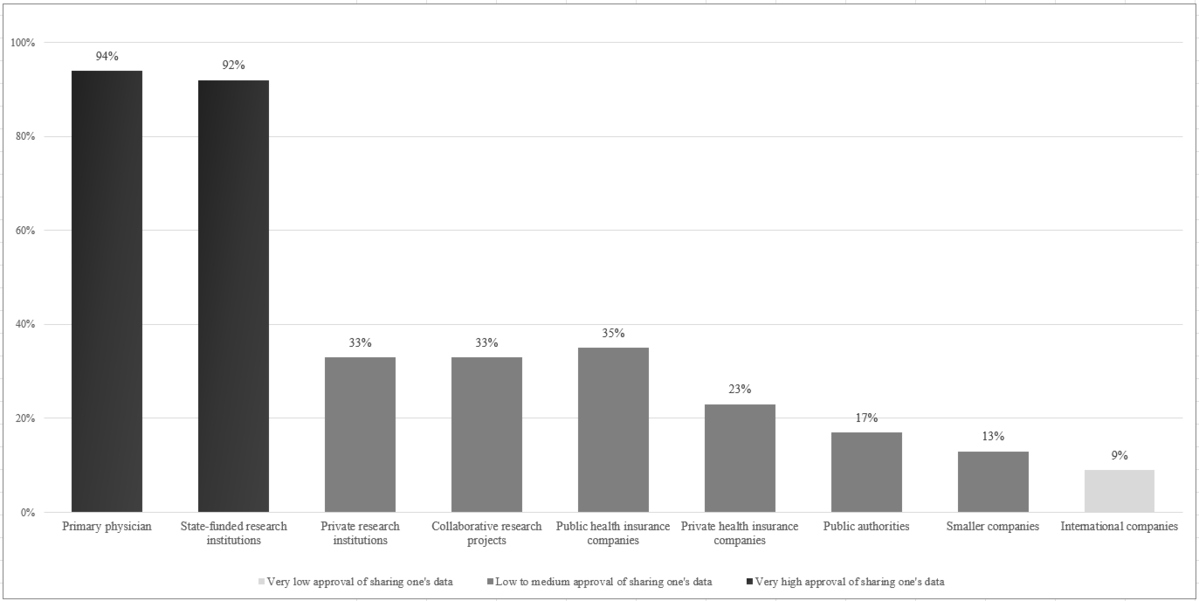

Supplement: Multimedia Appendix 4 [file cardio_v6i2e34959_app4.png]
